# SNP Discovery Using Next Generation Transcriptomic Sequencing in Atlantic Herring (*Clupea harengus*)

**DOI:** 10.1371/journal.pone.0042089

**Published:** 2012-08-07

**Authors:** Sarah J. Helyar, Morten T. Limborg, Dorte Bekkevold, Massimiliano Babbucci, Jeroen van Houdt, Gregory E. Maes, Luca Bargelloni, Rasmus O. Nielsen, Martin I. Taylor, Rob Ogden, Alessia Cariani, Gary R. Carvalho, FishPopTrace Consortium, Frank Panitz

**Affiliations:** 1 Molecular Ecology and Fisheries Genetics Laboratory, School of Biological Sciences, College of Natural Sciences, Bangor University, Bangor, Gwynedd, United Kingdom; 2 Food Safety, Environment & Genetics, Matís, Reykjavík, Iceland; 3 National Institute of Aquatic Resources, Technical University of Denmark, Silkeborg, Denmark; 4 Department of Comparative Biomedicine and Food Science, University of Padova, Legnaro, Italy; 5 Laboratory of Biodiversity and Evolutionary Genomics, Katholieke Universiteit Leuven, Leuven, Belgium; 6 Department of Molecular Biology and Genetics, Faculty of Science and Technology, Aarhus University, Tjele, Denmark; 7 TRACE Wildlife Forensics Network, Royal Zoological Society of Scotland, Edinburgh, United Kingdom; 8 Department of Experimental and Evolutionary Biology, University of Bologna, Bologna, Italy; University of Iceland, Iceland

## Abstract

The introduction of Next Generation Sequencing (NGS) has revolutionised population genetics, providing studies of non-model species with unprecedented genomic coverage, allowing evolutionary biologists to address questions previously far beyond the reach of available resources. Furthermore, the simple mutation model of Single Nucleotide Polymorphisms (SNPs) permits cost-effective high-throughput genotyping in thousands of individuals simultaneously. Genomic resources are scarce for the Atlantic herring (*Clupea harengus*), a small pelagic species that sustains high revenue fisheries. This paper details the development of 578 SNPs using a combined NGS and high-throughput genotyping approach. Eight individuals covering the species distribution in the eastern Atlantic were bar-coded and multiplexed into a single cDNA library and sequenced using the 454 GS FLX platform. SNP discovery was performed by *de novo* sequence clustering and contig assembly, followed by the mapping of reads against consensus contig sequences. Selection of candidate SNPs for genotyping was conducted using an *in silico* approach. SNP validation and genotyping were performed simultaneously using an Illumina 1,536 GoldenGate assay. Although the conversion rate of candidate SNPs in the genotyping assay cannot be predicted in advance, this approach has the potential to maximise cost and time efficiencies by avoiding expensive and time-consuming laboratory stages of SNP validation. Additionally, the *in silico* approach leads to lower ascertainment bias in the resulting SNP panel as marker selection is based only on the ability to design primers and the predicted presence of intron-exon boundaries. Consequently SNPs with a wider spectrum of minor allele frequencies (MAFs) will be genotyped in the final panel. The genomic resources presented here represent a valuable multi-purpose resource for developing informative marker panels for population discrimination, microarray development and for population genomic studies in the wild.

## Introduction

Population genomic approaches have been revolutionised by Next Generation Sequencing (NGS) technologies such as 454 (Roche) and Illumina sequencing. These developments facilitate genome-wide analyses of genetic variation across populations of non-model organisms [1], [2], allowing a range of evolutionary questions to be investigated effectively for the first time. Marine fishes are excellent model systems for studying adaptation due to their large geographic ranges that frequently encompass strong environmental gradients and their large population sizes that increase the relative strength of selection over drift [3]. Moreover, many marine fishes are under extreme anthropogenic pressure and there is an urgent need for genomic tools to identify population structure and boundaries to allow effective management [4]. Additionally the forensic identification of fish and fish products throughout the food processing chain from net to plate would assist in the fight against Illegal, Unreported and Unregulated (IUU) fishing, currently a priority for the European Union [5] and globally [6]. SNPs are the optimal marker for this type of application, but large SNP panels are currently available for few marine fish species (e.g. Atlantic cod (*Gadus morhua*) [7]; European hake (*Merluccius merluccius*) [8]). Thus, the development of genomic resources for marine fish is urgently required for evolutionary, conservation and management perspectives.

The strategy used for SNP development in non-model organisms is dependent on the availability of genomic information from closely related species. If such resources are available, PCR amplicons (homologous to regions in the reference genome) can be sequenced and SNPs identified (however, these are intrinsically limited in the number of SNPs that can be identified). Without a reference genome, three principal strategies for genome-wide SNP discovery can be applied; whole genome sequencing and assembly, genome complexity reduction and sequencing methods (e.g. RRL and RAD-seq) and cDNA sequencing (RNA-seq). While whole genome sequencing has now been completed for species with large complex genomes (for example: panda (*Ailuropoda melanoleura*) [9]; cacao (*Theobroma cacao*) [10]), this remains outside the scope of most studies, as in general the *de novo* assembly of larger, repeat-rich or polyploid genomes requires additional information (e.g. physical BAC maps or paired-end libraries) and extensive bioinformatic capacity in order to build the large, computationally intensive, structured sequence scaffolds [11]. Genomic libraries which sequence a small fraction of the genome (typically 3–5%) require a high level of coverage for contig assembly and detection of SNP variants (see [12]–[14] for applications). Deep sequencing of cDNA libraries provides an attractive approach to achieve the high sequence coverage needed for *de novo* contig assembly and SNP prediction, as only a small percentage of the genome is accounted for by the transcriptome. Another advantage of transcriptome sequencing is the information produced concerning functional genetic variation in specific genes which may be under selection; these can then be targeted to evaluate gene expression profiles. The ability to examine both neutral variation and genomic regions under selection provides researchers with unprecedented tools for understanding local adaptation of wild populations at the molecular level.

Atlantic herring (*Clupea harengus*) is an abundant and ecologically highly diverse species, occurring with a more or less continuous distribution in the North-Atlantic benthopelagic zone. Habitats are distributed across highly diverse environments, from temperate (33°N) to arctic (80°N) and at salinities from oceanic (∼35 ppt) to brackish (down to 3 ppt). In spite of its large ecological range, studies using “neutral” microsatellites have unanimously reported weak population differentiation that is statistically significant only on regional scales [15]–[17]. However, despite relatively high levels of gene flow among populations, evidence of local adaptation has been identified in the Atlantic herring in the Baltic Sea using microsatellite loci [18], [19]. Therefore it is expected that analyses with transcriptome-wide coverage applying hundreds of markers associated with adaptive and neutral variation will provide novel insights into the role of selective and demographic processes in shaping population structure.

We describe transcriptome-based SNP development in Atlantic herring using a Roche 454 GS FLX (hereafter 454) sequencing approach. Our aim was three-fold; 1) to develop a SNP assay exhibiting minimal ascertainment bias across east Atlantic populations, 2) to test the applicability of *in silico* SNP detection utilizing a combined SNP screening and validation approach as a cost efficient way of obtaining population genomic resources, and 3) to establish a transcriptome resource for tissue-specific gene expression profiling and microarray development. We present, to our knowledge, one of the first studies describing SNP discovery in a non-model marine fish based on transcriptome sequencing using NGS.

## Materials and Methods

### cDNA Library Construction and 454 Sequencing

**Figure 1:**
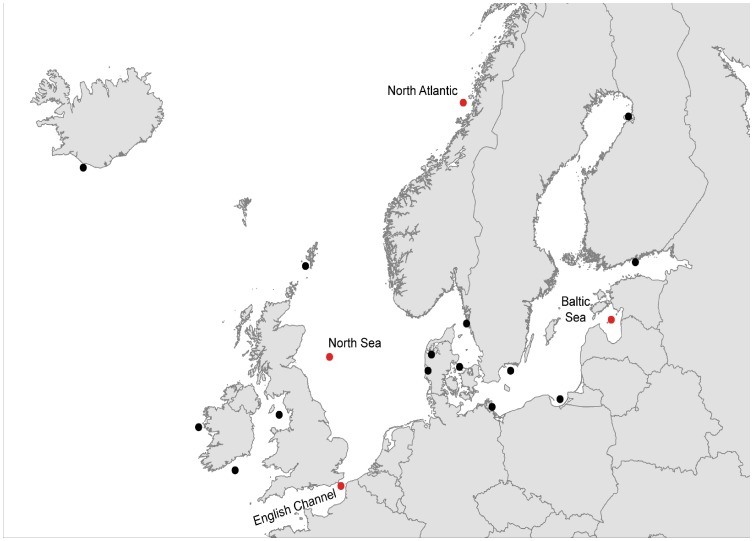
Location of the 18 samples used in this study. The eight sequenced ascertainment individuals (2 per location) came from the four sampling sites denoted in red.

SNP development was based on muscle samples from eight fish collected from four locations from across the eastern Atlantic (Figure 1). These locations were chosen to maximise geographic coverage and environmental differences, thereby minimising potential ascertainment bias. Approximately 5g of muscle tissue was taken from each of two individuals (male and female) from each location and immediately placed in RNAlater (Invitrogen) and after 12 hours at 4°C, were stored at −80°C. Total RNA was extracted using the RNeasy Lipid Tissue Mini Kit (Qiagen). The Oligotex mRNA Mini Kit (Qiagen) was used to isolate mRNA, and non-normalised cDNA was synthesized using the SuperScript Double-stranded cDNA Synthesis Kit (Invitrogen). A multiplex sequencing library was prepared by pooling equal amounts of cDNA from all eight individuals, where two specific 10-mer barcoding oligonucleotides were ligated to each individual sample to allow post-sequencing identification of sequences (modified from [20]). High-throughput sequencing was performed on a 454 sequencer according to the manufacturers’ protocol.

### Sequence Processing and Assembly

Sequences were first de-multiplexed using the barcoding tags (sfffile tool, Roche 454 analysis software) and sorted by sample. Mitochondrial sequences were removed from the data set by mapping the reads against the Atlantic herring mitochondrial genome (Genbank accession NC_009577 [21]) using the Roche 454 gsMapper software. RepeatMasker [22] was used to identify and mask repetitive and low complexity regions within the reads by using the zebrafish (*Danio rerio*) repeat library. Reads were cleaned for short sequences (<50 bp) and low quality regions using SeqClean (http://compbio.dfci.harvard.edu/tgi/software/). Sequence clustering was performed in two steps; initial clustering was performed using CLC Genomics Workbench (CLCbio, Denmark), the resulting ace file sequences were then assembled ‘per contig’ in CAP3 [23]. The consensus sequences for the contigs produced by this assembly were then used as a reference for mapping reads in the subsequent *in silico* SNP detection.

### SNP Detection

To identify candidate SNPs, all contig specific reads from the CAP3.ace files were re-mapped onto the consensus sequence and candidate SNPs were identified using GigaBayes [24]. This program scans each position of the assembly for the presence of at least two SNP alleles and calculates the probability of a given site being polymorphic using a Bayesian approach. No insertion or deletion variants (InDels) were considered and the polymorphism rate was set to 0.003. A minimum contig depth of four reads covering the polymorphic site and a minimum of two reads for the rare allele were required for a site to be considered as a putative SNP. All contigs containing SNPs were filtered to remove instances in which the alternative allele of the SNP was only identified in a single individual, as these may either represent false positives or may lead to strong ascertainment bias.

### Microsatellite Sequence Screening

Microsatellites are an important resource for smaller scale studies in population genetics, microsatellites within expressed genomic regions have been shown to produce clearer genotyping results as there are fewer null alleles and stutter bands [25], [26]; therefore the contig library developed here was screened to detect repeat regions. Assembled contigs were screened for microsatellite repeats using MsatCommander [27] a Python program which locates microsatellite repeats (di-, tri-, tetra-, penta-, and hexa-nucleotide repeats) within fasta-formatted sequences or consensus files. MsatCommander then uses Primer3 [28] to screen sequences containing microsatellite loci for high-quality PCR primer sites within the flanking regions for ‘potentially amplified loci’ (PALs [29]).

### Contig Annotation

Contigs were annotated using the Basic Local Alignment Search Tool (BLAST) against multiple sequence databases. Blastn searches (E-value cut-off <1.0 E^−5^) were conducted against all annotated transcripts of *Gasterosteus aculeatus*, *Tetraodon nigroviridis*, *Oryzias latipes, Takifugu rubripes*, *Danio rerio* and *Homo sapiens* available through the Ensembl Genome Browser, and against all unique transcripts for *D. rerio*, *H. sapiens*, *O. latipes*, *T. rubripes*, *Salmo salar*, and *Oncorhynchus mykiss* in the NCBI UniGene database. Blastx searches were conducted (E-value cut off <1.0 E^−3^) against the UniProtKB/SwissProt and UniProtKB/TrEMBL databases. Lastly Blastx searches were performed against all annotated proteins from the transcriptomes of *G. aculeatus*, *T. nigroviridis*, *O. latipes, T. rubripes*, *D. rerio* and *H. sapiens* available through the Ensembl Genome Browser.

To predict the effect of the mutation underlying each SNP at the amino acid level, a pipeline was developed to predict the reading frame for each SNP-containing contig. All contigs containing SNPs were first blasted against six peptide sequence databases (Ensembl genome assembly for *G. aculeatus*, *T. nigroviridis*, *O. latipes, T. rubripes, D. rerio* and the Swissprot database*)* using the Blastx function (E-value cut-off <1.0 E^−3^). For each SNP containing contig the best match was selected and the aligned sections of the query were saved. Subsequently, two 121 bp sequences per SNP (i.e. 60 bp up/down-stream of the SNP position, one sequence for each allele) were produced, these were used in a Blastx analysis against the file retrieved from the peptide sequences (E-value cut-off <1.0 E^−10^), and were then compared to determine if the SNP represented a synonymous or non-synonymous mutation.

### Selection of Candidate SNPs for Genotyping Assay

SNPs were validated following an *in silico* protocol, aimed at minimising validation costs, whilst also minimising subsequent locus dropout. SNP selection was based on the results from the Illumina Assay Design Tool, detection of putative intron-exon boundaries within the flanking regions of candidate SNPs, and a visual evaluation of the quality of contig sequence alignments. The SNPScore from the Illumina Assay Design Tool (referred to as the Assay Design Score/ADS) utilises factors including template GC content, melting temperature, sequence uniqueness, and self-complementarity to filter the candidate SNPs prior to further inspection. The Assay Design Score (assigned between 0 and 1) is indicative of the ability to design suitable oligos within the 60 bp up/down-stream flanking region, and the expected success of the assay when genotyped with the Illumina GoldenGate chemistry. Following the Illumina guidelines, all SNPs with a score below 0.4 were discarded; SNPs with a score above 0.4 were accepted, with SNPs scoring above 0.7 being used preferentially.

The prediction of intron-exon boundaries within the SNP flanking regions (60 bp up/down-stream of SNP position) was performed using two approaches. The first directly compared SNP-containing contigs against five high quality reference genomes for model fish species (Ensembl genome assembly for *G. aculeatus*, *T. nigroviridis*, *O. latipes, T. rubripes* and *D. rerio; s*ee Figure S1, left pipeline), using the Blastn option (E-value cut-off 10^−5^). Blast results were then parsed via a custom Perl script considering alignment length, start and end point of the alignment to determine the best positive match (further details of the Perl script and workflow are available from the authors on request). If the 60 bp on both sides of the SNP were present in the alignment, the candidate SNP was considered to be contained within a single exon; otherwise an intron-exon boundary was assumed to be present within the 121 bp assay design region. SNPs were then assigned to one of three categories either having, or not having an intron-exon boundary predicted within the flanking region, or as not returning a significant match against any of the five blasted fish genomes. In the other approach, the likelihood of a positive match and the reliability of intron-exon boundary predictions were increased, with SNP-containing contigs used as a query in a Blast search (blastn, E-value cut-off 10^−5^) against the corresponding transcriptome of the same five reference databases (see above). If the blast search produced a positive result, the matching transcript was downloaded from the Ensembl database, and blasted against its own genome sequence (see Figure S1, right pipeline). Within the downloaded sequence, the nucleotide position corresponding to the candidate SNP in the Atlantic herring sequence was identified based on the start and end positions of the alignment between the original contig and the Ensembl transcript. Using the projected SNP position, the flanking regions were again classified as being located on a single exon, disrupted by an intron, or not having a significant match. Results from the two approaches were compared to obtain a consensus estimate for the likelihood of an intron-exon boundary occurring within the 121 bp assay for each of the candidate SNPs.

Finally, the remaining candidate SNP contigs were visually evaluated using clview (clview; http://compbio.dfci.harvard.edu/tgi/software/) in order to rank putative SNPs within and among contigs. This was assessed by considering the overall quality of the assembly, the depth and length of alignments, and the number of mismatch sites flanking the SNP. This step was included to increase the likelihood of excluding incorrectly identified SNPs (for example; regions with alternative splicing or erroneous clustering of paralogous sequences). Within each contig, one or two SNPs receiving the highest quality score were considered for further validation (see below).

### SNP Validation

Following the pipeline described above, 1,536 high scoring candidate markers were chosen for validation by high throughput genotyping assay. DNA was extracted from fin clips for 626 fish sampled from eighteen sites across the species range in the eastern Atlantic, including twenty fish from each of the four SNP discovery populations (Figure 1). The quality and quantity of DNA was checked using a Nanodrop spectrophotometer, and all samples were standardised to 70 ng/µL. Genotyping was performed using the Illumina Golden Gate platform [30], and was visualised using Illumina’s GenomeStudio data analysis software (1.0.2.20706, Illumina Inc.). Only SNP assays showing clear genotype clustering, and individual samples with a call rate above 0.8 were considered for further analysis.

### Cross-species Amplification

To assess the utility of developed markers in related species, two species identified from a consensus phylogeny [31], the sister species; Pacific herring (*C. pallasii*) and a more distantly related species; anchovy (*Engraulis encrasicolus)* were genotyped for the full 1,536 SNP panel.

### Statistical Analyses

To assess the predictive value and utility of the different parameters used in the *in silico* SNP validation pipeline, a binomial logistic regression analysis was conducted. Two categorical variables (*Conversion* and *Polymorphism*) were evaluated which describe the outcome of the SNP assay validation; these are expected to depend on a range of candidate predictor variables (see below). *Conversion* was scored by assigning all 1,536 genotyped SNP assays as either failed (score  = 0) or successfully amplified and clustered (score  = 1). *Polymorphism* assigned all the successfully amplified SNP assays into monomorphic (0) or polymorphic (1) categories. Nine variables were then assessed for their predictive value in determining SNP assay conversion and polymorphism: i) number of ascertainment panel individuals supplying sequence reads at the SNP position, ii) number of sequences aligned under SNP position, iii) number of sequences with the minor allele, iv) frequency of sequences with minor allele, v) number of ascertainment individuals with the minor allele, vi) Illumina Assay Design Score (ADS), vii) outcome of the intron-exon boundary pipeline (scored as SNP assay being within a single exon, interrupted by an intron or as having no BLAST match), viii) number of reference species supporting findings from the intron-exon pipeline, and ix) neighbourhood sequence quality (determined by the number of mismatches in the flanking region alignment). To statistically test the predictive effect of the above variables for both *Conversion* and *Polymorphism* a two-step binomial logistic regression analysis was used as implemented in SPSS v12.0. All variables were included in the initial model, and a backward stepwise deletion approach was used for optimisation, in which the least informative variable is removed sequentially until only significantly contributing variables remain. A Wald χ^2^ statistic was used to estimate the relative contribution from each remaining parameter.

For the successful polymorphic assays global values of observed (H_O_) and expected (H_E_) heterozygosity were estimated for 20 individuals from each of the four ascertainment populations (Figure 1) using GenAlEx 6.4 [32]. For these same populations deviations from Hardy-Weinberg equilibrium (HWE) and evidence of linkage disequilibrium (LD) were explored using Genepop 4.0 [33]. Significance levels for HWE and LD tests were estimated using an MCMC chain of 10,000 iterations and 20 batches. *P*-values were adjusted for multiple tests by false discovery rate (FDR) correction following Benjamini & Yekutieli [34].

Lastly, ascertainment bias, resulting from the non-random exclusion of SNPs with a low Minor Allele Frequency (MAF) from the marker panel, may occur due to the small size (n = 8) of the ascertainment panel (compared to the whole population), and the limited geographical coverage (compared to the whole species range). When markers are then genotyped on a much larger sample of individuals the resulting ascertainment bias [35], [36] may affect the estimation of many evolutionary and population genetic parameters [2]. To assess the magnitude of a potential bias, the distribution of MAF in the marker panel was assessed across a large data set covering 18 locations across the Eastern Atlantic to check for an elevated non-random exclusion of SNPs with a low MAF. An un-biased SNP panel should exhibit an “L-shape” distribution of MAF categories indicating adequate representation of low MAF SNPs [37].

## Results

### 454 Sequencing

**Figure 2:**
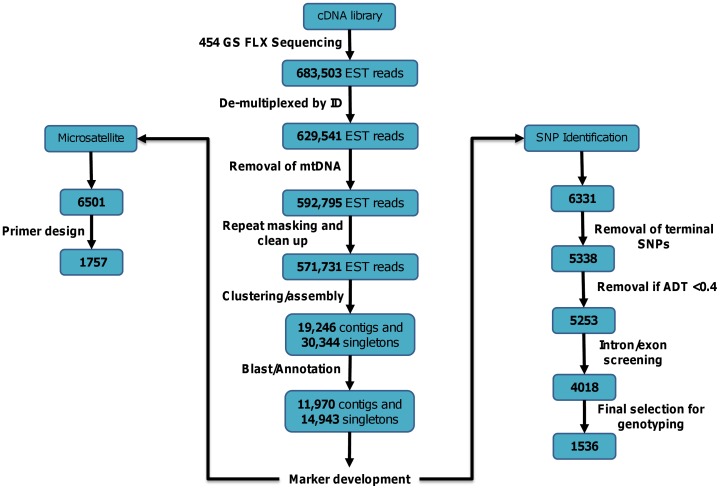
Schematic of transcript assembly and SNP detection pipeline. Schematic overview with numbers of reads, contigs and SNPs through the transcript assembly (centre) SNP detection (right hand side) and microsatellite detection (left hand side) pipelines (see text for more details).

**Figure 3:**
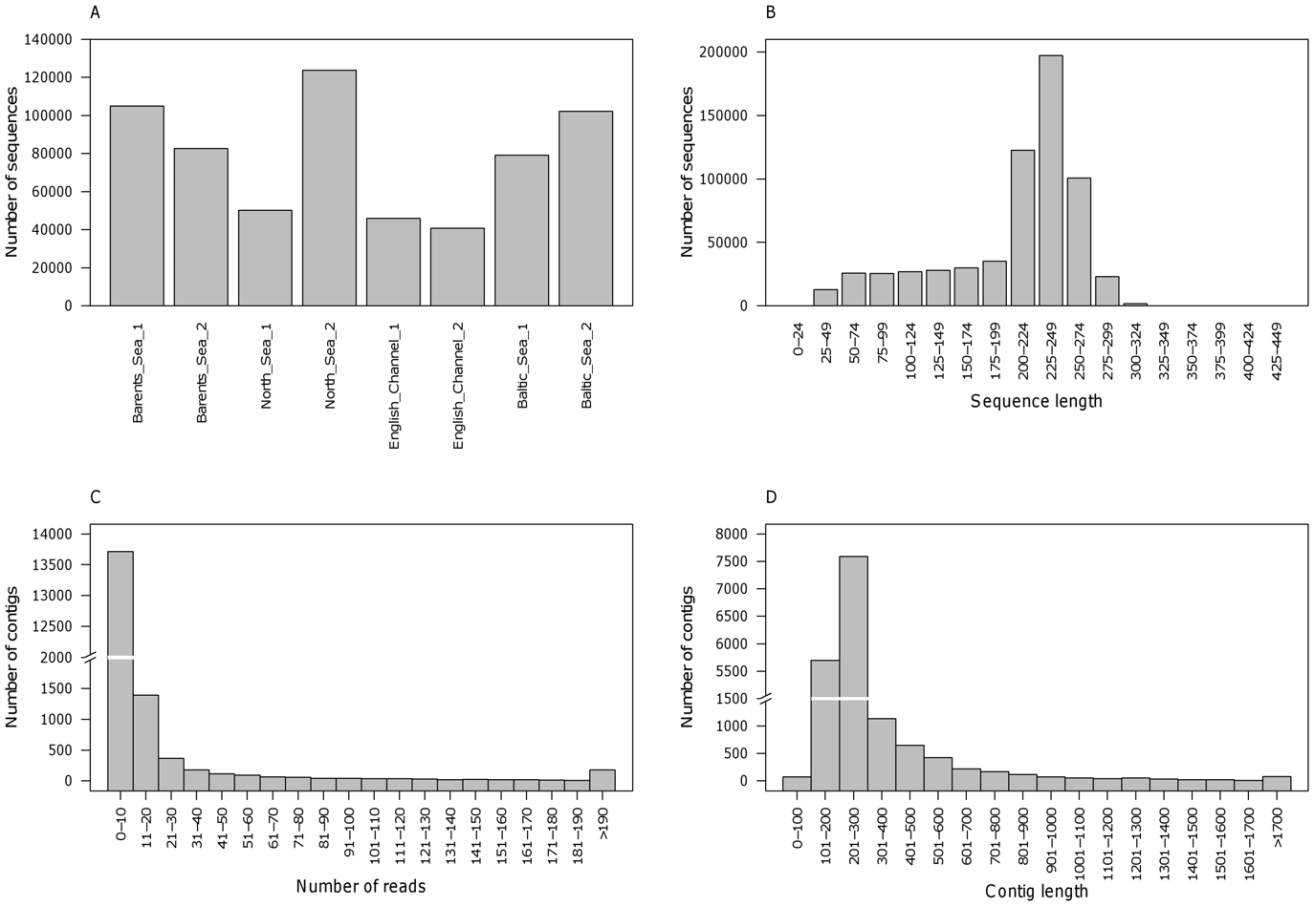
Summary of sequence data. A) number of sequences successfully barcoded for each of the eight ascertainment individuals; and for the combined data, B) sequence length, C) number of reads per contig and D) contig length.

Results for the sequencing and SNP discovery pipeline are illustrated in Figure 2. A total of 683,503 cDNA sequences were generated from the multiplexed Atlantic herring muscle library. The reads were de-multiplexed to assign reads to one of the eight sequenced individuals according to their barcoding tag. For 8% of the raw reads no barcoding tag was identified, while the remaining 629,541 raw reads (average read length: 205 bp, Figure 3B) contained the 5′ tag sequence and could be allocated to pools per sample per geographical region (Figure 3A). Geographic pools ranged from 86,731 (English Channel) to 187,554 (Barents Sea) sequences. All 454 sequence data has been submitted to the Sequence Read Archive (SRA) under the study accession number ERP001233 (http://www.ebi.ac.uk/ena/data/view/ERP001233).

### Sequence Processing and Assembly

Sequence cleaning and processing identified 5.8% of the assigned reads as having a match of at least 94% identity over 60 base pairs to Atlantic herring mitochondrial sequences and these were removed from the data set. RepeatMasker masked 1.9% of the dataset using the zebrafish repeat library. The SeqClean program removed a further 3.5% of the assigned reads due to low-complexity (n = 7,885), low quality (n = 169) or being below the minimum read length of 50 bases (n = 13,010). Lastly, some reads were trimmed, yielding a total of 571,731 reads for sequence clustering and assembly. Initially reads were clustered with CLC Genomics Workbench (CLCbio, Denmark), resulting in 16,456 clusters ranging from 200–400 bp. These were then individually re-assembled with CAP3 resulting in 19,246 contigs (some clusters produced by CLC were split into two or more contigs) and 30,344 singletons of which more than 50% could be annotated (Table 1). The majority of contigs consisted of less than 30 reads and ranged between 100–500 bp (Figure 3C-D).

**Table 1:** Number of contigs and singletons obtained and successfully annotated.

	Total	Annotated	%Annotated
**Contigs**	19,246	11,970	62.1
**Singletons**	30,344	14,943	49.2
**Total**	49,590	26,913	54.3

### SNP Detection and Annotation Results

SNP discovery with GigaBayes detected 6,331 putative SNPs in 1,991 separate contigs. The primary annotation of contig sequences is summarized in Table 1 and in more detail in Table S1.

### Selection of Candidate SNPs for Genotyping Assay

From the 6,331 predicted SNPs, 993 (15.6%) were located in the terminal region of the contigs and did not have the required minimum of a 60 bp flanking region to design oligos for the GoldenGate array (Figure 2). Of those remaining, 85 SNPs (1.3%) scored below the minimum value (<0.4) recommended for primer design and were not considered. 4,104 SNPs (76.8%) had high Assay Design Scores (between 0.7–1.0) and 1,149 SNPs (21.5%) had acceptable Assay Design Scores (between 0.4–0.7), all 5,253 of these were taken forward to the next stage. Of the putative SNPs screened for potential intron/exon splicing sites within the flanking regions, 1,235 (23.5%) had putative intron/exon boundaries within the flanking regions, and so were rejected. The majority (3,052, 58.1%) had no matching BLAST hits, while just 966 (18.4%) had BLAST hits which suggested that there was no intron/exon boundary present (summarised in Figure 2).

### SNP Validation

From the full 1,536 panel of SNPs that were genotyped, 290 (19%) assays failed to amplify. Of the remaining 1,246 assays, 201 were monomorphic (false positives: 13%) 467 produced ambiguous clustering (30%) and 578 were polymorphic, equivalent to a conversion rate of 38%. From these 578 SNPs an open reading frame was obtained for 270 of the respective 121 bp sequences (SNP and 60 bp up/down stream), of which 66 were suggested be non-synonymous, and 204 to be synonymous, equivalent to a ratio (non-synonymous/synonymous) of 0.32 (Table S2).

Results on the predictive value of the SNP selection parameters for assay conversion (i.e. for successful amplification) show that inclusion of all of the predictor variables (see methods) marginally improves model-fitting (χ^2^ = 18.520, d.f. = 9, p<0.030). When using backward stepwise deletion of predictor variables, the Assay Design Score and number of ascertainment individuals with the minor allele were identified as the only significant predictors of assay conversion, but only the Assay Design Score showed the expected positive correlation with conversion rate (Table 2). The binomial logistic regression analysis on the polymorphic status of all successfully amplifying assays showed that when all predictor variables were included, the overall model fit was not significant (χ^2^ = 11.554, d.f. = 9, p = 0.240). However, neighbourhood sequence quality had a significant negative correlation with polymorphism. As before a backward stepwise deletion approach was used and this reduced the significantly contributing predictors to the number individuals in the ascertainment panel with the minor allele and the neighbourhood sequence quality which, as expected, respectively showed positive and negative correlation with SNP polymorphism (Table 3).

**Table 2:** Results for SNP detection variables for predicting SNP assay conversion following a backward stepwise elimination procedure.

	B^a^	Wald^b^	df	P^c^
***Asc_ind*** d	−0.165	4.67	1	**0.031**
***ADS*** e	0.763	4.785	1	**0.029**
**Constant**	−0.378	1.464	1	0.226

a Regression coefficient for individual variable.

b Wald χ^2^ statistic.

c associated probability.

d Number of ascertainment individuals with the minor allele.

e Assay Design Score. Significant p-values are shown in bold.

**Table 3:** Results for SNP detection variables for predicting SNP assay polymorphism following a backward stepwise elimination procedure.

	B^a^	Wald^b^	df	P^c^
***Asc_ind*** d	0.249	2.965	1	0.085
***NSQ*** e	−0.111	7.321	1	**0.007**
**Constant**	0.935	21.137	1	0.000

a Regression coefficient for individual variable.

b Wald χ^2^ statistic.

c associated probability.

d Number of ascertainment individuals with the minor allele.

e Neighbourhood Sequence Quality. Significant p-values are shown in bold.

**Figure 4:**
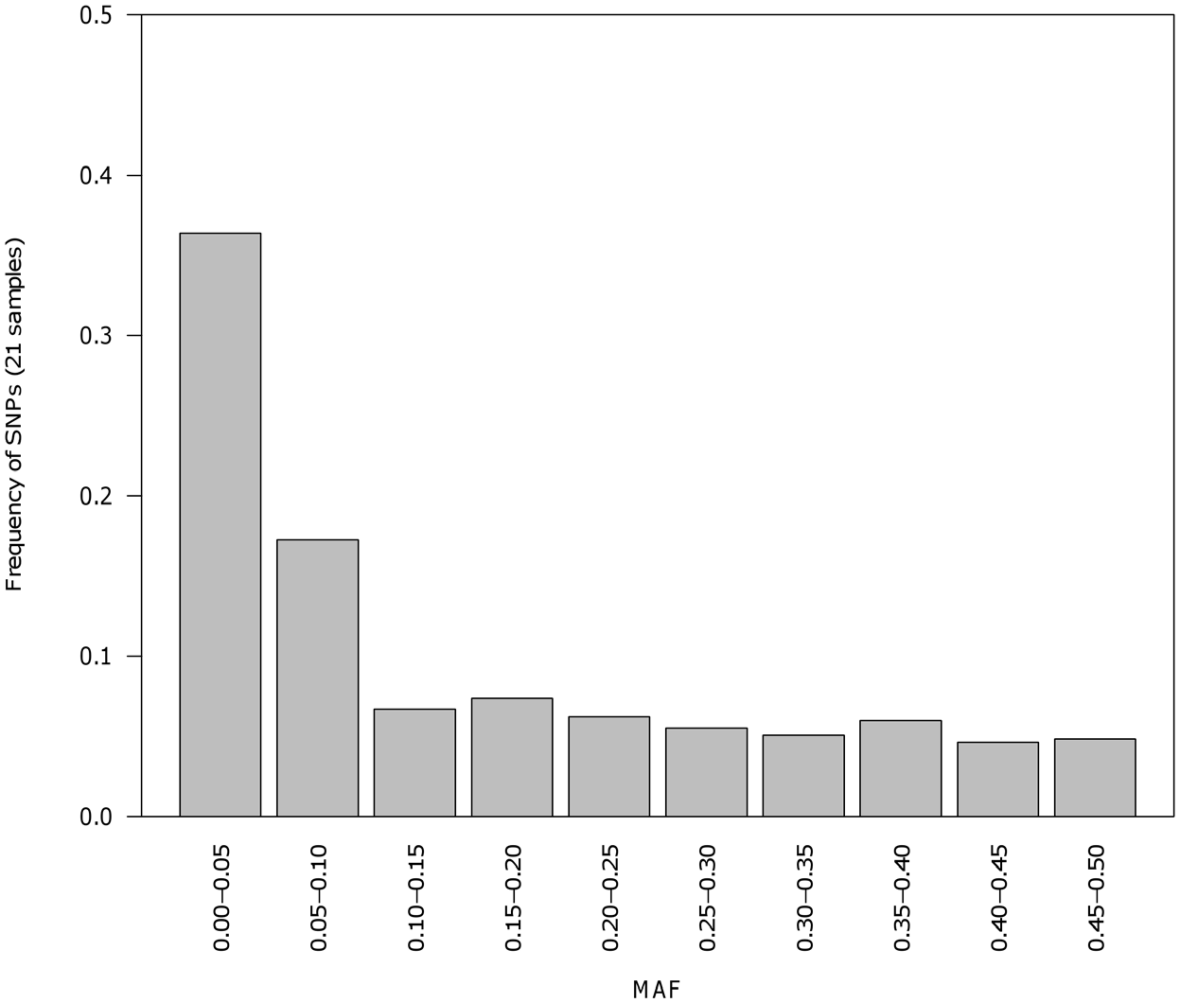
Minor allele frequency (MAF) distribution. The distribution of the MAF in 578 SNPs typed in 18 populations across the eastern herring distribution.

Estimates of *H_O_* and *H_E_* across the four ascertainment samples ranged from 0.00–0.63 (mean 0.18) and 0.00–0.50 (mean 0.18), respectively (Table S2). Observed heterozygosity within the four ascertainment populations revealed similar levels of diversity to the 18 sampled locations used for the SNP validation [38]. Tests for deviation from HWE for each locus and population revealed 43 out of 1,249 performed tests (3.4%) with significant deviations from HWE before correction for multiple tests. These tests were distributed among all four populations and across 35 loci. Eight tests distributed across three populations and seven loci retained significance following correction for multiple tests (α = 0.05). Due to the presence of monomorphic loci in the four ascertainment samples, 229,094 tests for LD were performed of which 352 remained significant after correction for FDR (α = 0.05). Of these, 14 pairs were significant in more than one of the four populations but in all cases SNPs originated from different contigs suggesting lack of close physical linkage. SNP frequency distributions of MAF categories in the full panel of 18 samples indicated little bias due to non-random selection of high frequency SNPs (Figure 4).

### Cross-species Amplification and Microsatellite Detection

The majority (99%) of the 578 markers identified as polymorphic in Atlantic herring also amplified in Pacific herring, but only 12% exhibited more than one allele. Only about 10% of the 578 SNPs amplified in anchovy, and of these, only ten loci exhibited polymorphism.

MsatCommander detected 6,501 microsatellites with a repeat length of between two and seven bases with four or more repeat units in 3,741 contigs (Table 4). 27% of the microsatellites had sufficient suitable flanking sequence to enable the design of primers. Details of the microsatellites (number and type of repeat, primers, Tm and %GC) are listed in Table S3.

**Table 4:** Type and number of repeats of the microsatellites detected in the herring contigs using Msatcommander.

Type of repeat	Number of repeats					Total
	4–9	10–14	14–19	>19	Maximum	
Dinucleotide	4418	505	193	175	75	5291
Trinucleotide	829	35	9	2	36	875
Tetranucleotide	202	13	3	12	31	230
Pentanucleotide	43	1	1	0	17	45
Hexanucleotide	57	2	0	1	21	60
Total	5549	556	206	190	-	6501

## Discussion

This study demonstrates the *de novo* discovery of 6,331 putative SNPs based on 454 transcriptome sequencing of eight individuals covering the Northeast Atlantic distribution of the Atlantic herring. Of particular interest in the approach is the single validation and genotyping step, disposing with the traditional step of testing each SNP for amplification prior to large scale genotyping (e.g. [39], [40]). The data generated in this study constitutes a new resource for genetic analysis in Atlantic herring significantly increasing the number of known transcripts as well as novel SNP and microsatellite markers.

### Sequence Assembly and SNP Detection

For next generation sequencing to be successfully applied to the development of genetic resources in non-model organisms, methodological issues must be addressed to optimise the procedures for each project. SNPs can be genome- or transcriptome derived and, in the latter case, selected from more abundant or rarer expressed transcripts; in addition, marker development is influenced by sequence depth and contig length due to the sequencing platform chosen and the complexity of the hypothesis to be investigated (i.e. smaller number of SNPs required for species identification analysis as compared to population genetic studies). The choice of sequencing platform should reflect the objective of a given study. While longer reads (e.g. 454 sequencing) are expected to improve contig assembly, more, but shorter, reads (e.g. Illumina sequencing) may be preferable in order to reduce detection of false positive SNPs from higher alignment depth, especially when an existing reference sequence is available. This study took advantage of the longer read lengths obtained with 454 sequencing in a *de novo* assembly of a reference scaffold for SNP discovery in herring. The clustering and assembly step is critical for SNP mining as it generates the reference for variant detection by mapping reads to the contig. Therefore, the absence of a reference genome or transcriptome poses a challenge for assessing the ‘correctness’ of a contig assembly, as potential mis-assemblies of sequence due to homologous or paralogous genes cannot be directly verified by back-mapping to the species-specific genome. Generally, cluster assembly with overly stringent parameters will lead to splitting sequences belonging together into more contigs, resulting in a higher number of shorter contigs with lower coverage depth. Whilst applying criteria that are overly relaxed will assemble reads from related genes or gene families into single contigs, resulting in a lower numbers of contigs that have a higher sequence depth, however this increases the likelihood of misidentifying polymorphisms between paralogous sequence variants (PSVs) as SNPs. Additionally, as no genome reference is available for Atlantic herring, the occurrence of PSVs cannot be assessed, this was probably the cause for the majority of ambiguous clustering that was subsequently seen in the SNPs.

For the SNP detection, the low sequence depth of the majority of contigs (Figure 3C) required relatively low criteria to be set (i.e. depth: four reads, redundancy: two observations of the minor allele). However, these low thresholds together with the sequencing of eight ascertainment individuals spanning the entire northeast Atlantic distribution of herring resulted in minimal ascertainment bias due to exclusion of low MAF SNPs (Figure 4). One expected result of the low depth and redundancy parameters is, however, the low conversion rate from the inflated number of candidate SNPs (identified due to sequencing errors). The 454 platform-specific challenge of resolving homopolymeric regions may further have compromised SNP detection by reducing assembly quality or calling false SNPs within these regions [41], but such an effect could not be assessed here due to the lack of a known reference sequence.

The use of transcriptome sequencing in this study has resulted in only a few per cent of the total genome being covered, but at a relatively high sequencing depth, thus limiting sequencing costs while achieving the number of SNPs required for custom-designed SNP assays. Additionally, transcriptome sequencing provides information about tissue-specific genes and their expression profile, which can be used to develop further tools for gene expression studies such as oligonucleotide microarray or RNA-seq approaches.

### SNP Validation

The genotyping of 1,536 selected SNP assays performed with genomic DNA for a large panel of Atlantic herring samples from across the northeast Atlantic indicated that nearly 600 of the SNPs are polymorphic (37.6%). However, almost 49.3% of the candidate SNPs failed to work; due to either non-amplification (18.9%), false positives (monomorphic loci) (13.1%) or ambiguous clustering (17.3%). Despite our attempt to screen for potential intron/exon splicing sites within flanking regions of all candidate SNPs using available reference genomes, only 41.9% of all queries matched equivalent sequences in at least one of the reference species. Thus, the presence of undetected introns may have constituted a major cause for genotyping failure [42]. Moreover, candidate SNPs that appeared monomorphic in the large-scale screening might either be the result of false-positive predictions or could indicate real, rare SNPs not present in the samples tested [7]. The purely *in silico* SNP detection method presented in this study may have a relatively low conversion rate to validated SNPs when compared to other methods. However, this method is still extremely competitive given a limited resource for marker development, once the time and cost associated with designing and ordering hundreds of primers, running validation PCRs, and additional Sanger sequencing for validation are considered (e.g. [39], [40]). All of which would be in addition to the cost of genotyping the resulting 578 validated SNPs.

In order to reduce the number of erroneous SNP predictions, i.e. to increase the probability of an *in silico* detected SNP being a truly polymorphic site, further sequencing would lead to greater sequence depth of the contigs, allowing more stringent selection of SNP candidates. It has been shown for multiplexed re-sequencing that more than 90% of the variants can be detected correctly using next generation sequencing technologies when an average depth of at least 20 reads per base is achieved [43], [44]. Increasing the average sequence depth will also be advantageous for identifying SNPs from rarely expressed genes. Another interesting approach, recently described by Ratan *et al.* [45], suggests a method to call SNPs without a reference genome sequence. SNP calling is performed whenever new sequences are added; thus, sequencing continues only as long as needed to identify an adequate number of candidate SNPs. The method is reported to work even when the sequence coverage is not sufficient for *de novo* assembly. Additionally, the use of next generation sequencing for analysing a restriction enzyme-generated DNA library (RRL and in particular RAD sequencing, for reviews see [46], [47]) based on multiple tagged individuals now enables the fast discovery of thousands of SNPs in non-model organisms with no prior genome information [48], [49]. However, one downstream problem identified with RAD-seq is that transferring the SNPs onto a high-throughput genotyping platform is difficult without a reference genome, as the majority of SNPs identified do not have the 60 bp flanking sequenced required for assay design. This has to some extent been solved using Paired End RAD (RAD-PE)[50], however the bioinformatic approaches for SNP discovery in RAD-PE contigs are still limited. Additionally, while RRL/RAD-seq approaches eliminate the problems encountered with intron/exon boundaries that are associated with transcriptome sequencing, these methods only consider random fragments of the entire genome, whereas our transcriptome based pipeline specifically targets expressed genes with an increased likelihood for detecting SNPs (e.g. non-synonymous substitutions) associated with genomic regions under selection. Such non-neutral SNPs are expected to provide high discriminatory power at the population level and will constitute a valuable forensic tool in future applications [47], [51]. The combination of the coverage and SNP discovery rates obtained by RAD-seq, with the targeted reduction obtained by sequencing the transcriptome would potentially be a very powerful tool. However, it must be noted that due to the rapid rate of technical developments in the field, such as the increased read length and decreasing costs of existing platforms, and the potential of nano-sequencing technology, the best solution regarding platforms and methods to optimise the cost effectiveness for a specific application needs careful consideration.

When determining the predictive value of the SNP selection parameters for successful amplification of the *in silico* detected SNPs (*Conversion)*, as expected, a positive correlation was found with the Assay Design Score, i.e. the likelihood for designing successful primers around the SNP position. Unexpectedly, a negative correlation was found with number of ascertainment individuals for which the rare allele was observed, although the reasons behind this correlation are unclear. Overall, only very weak predictive variables for *Polymorphism* were identified, with only the neighbourhood sequence quality significantly explaining the negative correlation; as the number of mismatches in flanking regions increases, a predicted SNP is more likely to be a false positive. This increase in mismatches of an aligned region could be indicative of erroneous clustering, for example, PSVs or other sequences with differing genomic origin (this has for example also been seen for hake in a similar study [8]). The number of individuals with the minor allele in the ascertainment panel also showed a positive correlation with *Polymorphism.* While this parameter is less conclusive than for predicting *Conversion* rate, there is potentially a predictive role of this parameter for detecting true SNPs. Future SNP development efforts may reduce the false positive rate by applying relatively stringent thresholds for this variable (e.g. having at least 2 individuals with the minor allele represented in the SNP containing contig, although this will, of course, depend on the size of the ascertainment panel).

The two binomial logistic regression analyses were repeated with a reduced set of variables representing the strongest *a priori* candidates (the number of sequences aligned under the SNP position, the frequency of sequences with minor allele, the neighbourhood sequence quality, the Assay Design Score, and the outcome of the intron-exon boundary pipeline). This also allowed controlling for a potential bias from non-independent variables such as the two intron-exon and three minor allele related parameters. Results were largely congruent confirming Assay Design Score and neighbourhood sequence quality to be the most significant predictors of *Conversion* and *Polymorphism*, respectively.

The range of allele frequencies within the SNP panel suggests that the strategy of carefully selecting individuals to maximise the geographical, phenotypic and genetic diversity covered by the SNP development samples has been successful in minimising ascertainment bias.

### Cross Species Amplification and Microsatellite Detection

A high proportion of detected SNPs also amplified single PCR products in Pacific herring albeit with a low polymorphism rate, which is as expected due to their development from conserved genomic regions. However, due to the small sample size (n = 4), this number is likely to be downwardly biased and a much higher proportion of SNPs may in fact be polymorphic and therefore prove useful in this species. As expected from the phylogenetics of these species, the proportions of SNP amplification and polymorphism were lower in the anchovy. Additionally, our sequencing effort has led to the discovery of a large resource of microsatellite markers, 36% of which have primers successfully designed (Table S3). These include both neutral loci and loci that are physically linked to SNPs representing genomic regions that have been shown to be under directional selection [38]. Another attribute of multi-allelic microsatellite markers when studying adaptive genetic variation is the increased statistical power for detecting balancing selection compared to bi-allelic markers (such as SNPs, e.g. [52]), and also for applications such as parental assignment.

### Conclusion

Our approach of applying barcoding and multiplexing individuals for large-scale *in silico* mining of transcriptome sequences seems to be a very appropriate strategy to develop new SNP markers in non-model species as it does not require costly and time-intensive re-sequencing of target amplicons necessitating prior knowledge and availability of genome sequence information. However, the purely *in silico* based SNP detection comes with a trade off in the form of an expectedly lower conversion rate in the final genotyping assay [53]. The resultant resources will be of value in on-going analyses of population structuring and stock dynamics, assays of adaptive variation, and for enhancing the scope of microsatellite-based studies.

## Supporting Information

Figure S1 Analysis pipeline. The path on the left of the figure illustrates the pipeline for the genomic approach, where herring transcripts are directly compared with five reference genomes. The path on the right of the figure shows the pipeline for the transcriptomic approach, where herring transcripts are first compared to the transcriptome of the five reference species. Hits were then subsequently matched to the corresponding genomes of the same species (see text for more details).
(TIF)

Table S1 Number of contigs and singletons annotated using a range of fish and human reference resources and databases.
(XLSX)

Table S2 List of the 578 validated polymorphic SNPs found in this study, including the 120 bp flanking region, with the two SNP alleles in brackets. Also global estimates of observed (Ho) and expected heterozygosity (He) in the four ascertainment populations for each SNP. The S/NS column denotes whether a SNP was either synonymous (S) or non-synonymous (NS) with NA designating SNPs with no contig match in the BLAST search (see text for more details).
(XLSX)

Table S3 List of the microsatellites for which primers were successfully designed, along with up to 200 bases flanking sequence.
(XLSX)
