# Supplementary figures and images for: SNP Discovery Using Next Generation Transcriptomic Sequencing in Atlantic Herring (Clupea harengus)
Source: PLoS One. 2012 Aug 7;7(8):e42089. doi: 10.1371/journal.pone.0042089 (PMC3413699; doi:10.1371/journal.pone.0042089)

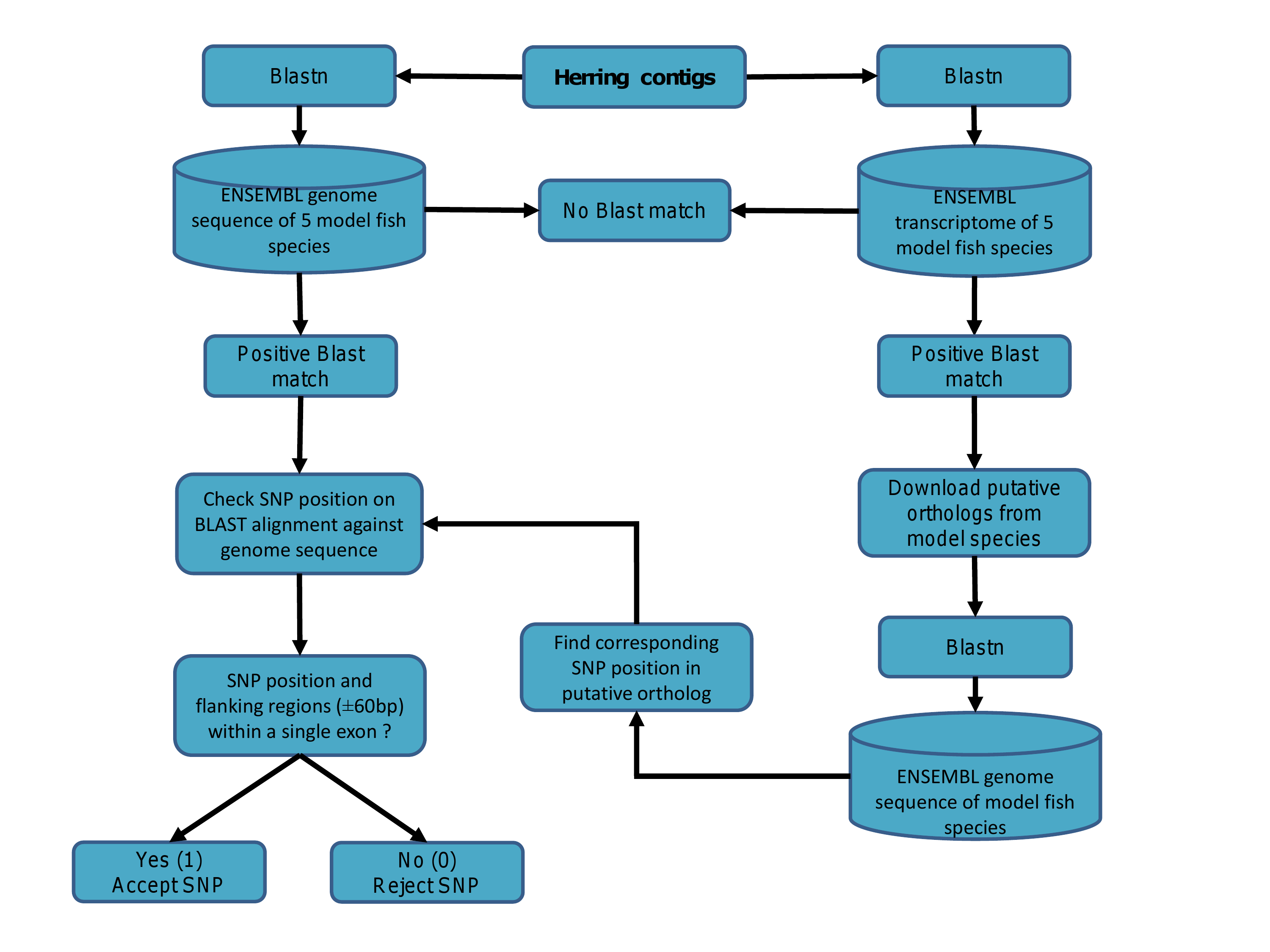

Supplement: Figure S1 — Analysis pipeline. The path on the left of the figure illustrates the pipeline for the genomic approach, where herring transcripts are directly compared with five reference genomes. The path on the right of the figure shows the pipeline for the transcriptomic approach, where herring transcripts are first compared to the transcriptome of the five reference species. Hits were then subsequently matched to the corresponding genomes of the same species (see text for more details). (TIF) [file pone.0042089.s001.tif]
